# Upregulated expression of human neutrophil peptides 1, 2 and 3 (HNP 1-3) in colon cancer serum and tumours: a biomarker study

**DOI:** 10.1186/1471-2407-5-8

**Published:** 2005-01-19

**Authors:** Jakob Albrethsen, Rikke Bøgebo, Steen Gammeltoft, Jesper Olsen, Benny Winther, Hans Raskov

**Affiliations:** 1 Department of Clinical Biochemistry, Glostrup Hospital, 2600 Glostrup, Denmark; 2 Surgical department D, Glostrup Hospital 2600 Glostrup, Denmark; 3 Colotech ltd., Copenhagen Science Park Symbion, Fruebjergvej 3, 2100 Copenhagen, Denmark

## Abstract

**Background:**

Molecular markers for localized colon tumours and for prognosis following therapy are needed. Proteomics research is currently producing numerous biomarker studies with clinical potential. We investigate the protein composition of plasma and of tumour extracts with the aim of identifying biomarkers for colon cancer.

**Methods:**

By Surface Enhanced Laser Desorption/Ionisation – Time Of Flight / Mass spectrometry (SELDI-TOF/MS) we compare the protein profiles of colon cancer serum with serum from healthy individuals and the protein profiles of colon tumours with normal colon tissue. By size exclusion chromatography, we investigate the binding of HNP 1-3 to high mass plasma proteins. By microflow we investigate the effect of HNP 1-3 on mammalian cells.

**Results:**

Human Neutrophil Peptides -1, -2 and -3 (HNP 1-3), also known as alfa-defensin-1, -2 and -3, are present in elevated concentrations in serum from colon cancer patients and in protein extracts from colon tumours. A fraction of HNP 1-3 in serum is bound to unidentified high mass plasma proteins. HNP 1-3 purified from colon tumours are lethal to mammalian cells.

**Conclusions:**

HNP 1-3 may serve as blood markers for colon cancer in combination with other diagnostic tools. We propose that HNP 1-3 are carried into the bloodstream by attaching to high mass plasma proteins in the tumour microenvironment. We discuss the effect of HNP 1-3 on tumour progression.

## Background

The diagnostic stage of colon cancer determines survival. Patients diagnosed with localized tumours have a 75% probability of 5 year survival, whereas patients diagnosed with distant metastases only have a 5–10 % probability of 5 year survival (reviewed in [1]). Recently a number of studies have been published in which Surface Enhanced Laser Desorption/Ionisation-Time Of Flight/Mass Spectrometry (SELDI-TOF/MS) has been applied to biological samples from patients with various forms of cancer [2-4] leading to the identification of protein markers with clinical potential. Here we present a SELDI-TOF/MS study of colon cancer serum and tumours. We show that the expression of Human Neutrophil Peptides -1, -2 and -3 (HNP 1 -3), also known as alfa-defensin-1, -2 and -3, is upregulated in the tumour microenvironment, as compared to normal colon tissue. This finding is reflected in serum. We find that HNP 1-3 is present in elevated concentrations in serum from patients diagnosed with tumours in the colon, as compared to serum from a healthy control group matched by age and gender. By size-exclusion analysis we add to the existing evidence that HNP 1-3 bind to high mass plasma proteins, explaining the presence of HNP 1-3 in serum. By microflow analysis, we show that HNP 1-3 purified from colon tumours are lethal to mammalian cells. The HNP 1-3 peptides are part of the defensin family of peptides (reviewed in [5-7]), which are a fundamental component of the immune system and have the capacity to kill / inactivate a broad range of pathogens. Defensins are also known to function as regulators of both the innate and the adaptive immune system. We discuss the possible effects of HNP 1-3 in the tumour microenvironment.

## Methods

### Biological samples

All biological samples were obtained by trained staff at Glostrup Hospital, Denmark. Written consent was obtained from all donators. Permission was obtained from the Danish Scientific Ethical Committee and the Danish Data Protection Agency.

### Tissue screening

Normal colon tissue samples and colon tumour samples were obtained from the removed fragment of the patient's colon after surgical treatment for colon cancer. Tissue samples were stored at -80°C until use. The protein content was extracted from the tissue: 100 mg tissue sample was thawed on ice and homogenised on a Wheaton Overhead Stirrer for 2 minutes at speed step 2, in 500 ul Lysis buffer (100 mM TRIS-HCl, pH 8.0, 9.5 M UREA, 2% CHAPS). The samples were centrifuged at 14,000 rpm for 10 minutes and the pellet was discarded (repeated twice). The tissue protein extracts were stored at -80°C until use. Pilot studies were performed on different chips (data not shown) and the NP20 (Normal Phase) (Ciphergen) chip was chosen for the tissue screening. NP20 chips were placed in Bioprocessor (Ciphergen) and pre-treated with 50 ul tissue binding buffer (50 mM TRIS-HCl, pH 8.0) for 5 minutes on shaker (250 rpm) (repeated twice). 5 ul tissue protein extract was diluted in 50 ul tissue binding buffer and incubated in Bioprocessor on NP20 chips for 40 minutes at room temperature on shaker (250 rpm). Spots were washed twice in 250 ul tissue washing buffer (50 mM TRIS-HCl, pH 8.0) for 5 minutes. The chips were air dried for 10 minutes, followed by treatment with two times 0.6 ul 100% sinapinic (SPA) matrix solution. SPA was obtained from Ciphergen in 5 mg aliquots and dissolved (150 ul MQ water, 150 ul acetonitrile, 1.5 ul Tri-Fluoro-acetic-Acid (TFA)) immediately before the screenings.

### Serum screening

Colon cancer serum samples were obtained from patients before surgical treatment. Normal serum was obtained from a group of healthy individuals matched by age and gender to the cancer patients. All serum samples were stored at -80°C until use. Serum pilot studies were performed on different chips to monitor the presence of HNP 1-3 in serum (data not shown). The immobilised metal affinity capture (IMAC30) chip was chosen for the actual screening and was pre-treated with nickel before analysis: 5 ul 100 mM NiSO4 were added to each spot and left on shaker (250 rpm) for 5 minutes (repeated twice). The chips were placed in Bioprocessor and incubated with 100 ul MQ for 5 minutes on shaker (250 rpm). Each spot was treated with 50 ul serum binding buffer (100 mM TRIS-HCl, pH 7.5, 500 mM NaCl, 0,1% Triton X-100) and left on shaker for 5 minutes (250 rpm). Serum samples were thawed on ice and 1 ul serum was diluted in 50 ul serum binding buffer and applied to spots and left on shaker (250 rpm) at room temperature for 40 minutes. The sample solution was removed and the spots were washed twice in 200 ul serum washing buffer (100 mM PBS, pH 7.4, 700 mM NaCl), followed by one wash in 200 ul MQ water. The chips were removed from the Bioprocessor and left to air dry for 20 minutes followed by treatment with two times 0.6 ul SPA.

Only freshly made matrix solutions were used and the instrument was calibrated daily. Cancer and normal samples were run side by side. The chips were analysed on a PBS II instrument (Ciphergen). All spectra in each screening were normalised based on total ion current.

### Purification and identification of HNP 1-3

100 ul protein extract from colon tumour tissue in tissue lysis buffer was loaded unto a RP-HPLC column (uRPC C2/C18 ST 4.6/100, Pharmacia Biotech, Flow rate: 0.5 ml/min, Fraction size: 0.5 ml) in buffer A (0.065% TFA in MQ-water) and proteins were eluted in a gradient of 0–100% buffer B (0.05% TFA in acetonitrile (ACN)). Elution of peptides was monitored by absorbance at 280 nm. All protein-containing fractions were analysed by Matrix Assisted Laser Desorption/Ionisation-Time of flight (MALDI-TOF) on the PBS II instrument: 1.5 ul fraction was incubated with 0.6 ul SPA on a Gold array (Ciphergen) and left to crystallise, followed by an additional treatment with 0.6 ul SPA and analysed in the PBSII instrument. The HNP 1-3 containing fraction (32% buffer B) was further purified on a peptide gelfiltration column (Superdex Peptide HR 10/30, Pharmacia Biotech, Flow rate 0.9 ml/min, Fraction size: 0.5 ml, Buffer: 50% ACN, 0.1 % TFA). Elution of peptides was monitored by absorbance at 280 nm and protein-containing fractions were again analysed by MALDI-TOF. Purified HNP 1-3 were identified by on-chip trypsin digestion: 10 ul of HNP 1-3 fraction was applied to an NP20 chip and left on shaker (250 rpm) at room temperature for 40 minutes. The solution was removed and the spot was washed twice with 10 ul water. In order to denature peptides prior to digestion, the chip was left on heating block (80°C) for 5 minutes. The chip was cooled on ice for 2 minutes. 10 ul trypsin digestion solution (0.01 ug/ul trypsin in 50 mM NH_4_HCO_3_, pH 8,0) was added, and the chip was left for 10 hours at 40°C in humidity chamber after which the chip was left to air dry for 20 minutes. 1 ul CHCA matrix (prepared as the SPA matrix solution) was added and the peptide map was analysed on the PBS II instrument. Peptide maps of trypsin autodiggest were used as controls. Identification was done with the PepIdent software on the Expasy server. For the reduction experiment, HNP 1-3 were first denaturation by heating (10 minutes at 80°C) followed by treatment with DTT (200 mM, 30 minutes at room temperature) and the peptides were incubated on an NP20 chip and analysed on the PBS II instrument.

### Size exclusion chromatography of HNP 1-3

50 ul colon cancer serum was loaded unto a peptide gelfiltration column (Superdex Peptide HR 10/30, Pharmacia Biotech, optimal separation range: 1 to 7 kDa, flow rate: 0.5 ml/min, fraction size: 0.5 ml, buffer: 10 mM Ammonium carbonate, pH: 8.0). Elution of peptides was followed by absorbance at 280 nm. All protein containing fractions were analysed by MALDI-TOF on PBS II (Ciphergen) as described above. Maximum signal intensity of 40 individual peaks was plotted as a function of elution volume and an approximate elution curve was calculated.

### Study of HNP 1-3 by microflow

For micro flow experiments, canine kidney cells (MDCK cells) were plated onto poly-d-lysine coated cover slips at a concentration 3000 cells/well, grown in Dilbeccoo's Modified Eagle Medium (DMEM) with 10% Fetal Bovine Serum (FBS) for five days with the result of confluent islands. Microflow was performed in an Eppendorf micromanipulator 5171 and transjector 5246 system mounted on a Leica DMIRBE inverted research microscope. Micro capillaries (borosilicate with filament, Sutter Instruments Company, Novato, California, USA) were pulled to an outer diameter of 0.85 nm on a Sutter P-97 Micropipette Puller. The dye-loaded cells were visualized by excitation at 470 nm and recorded at 509-nm emission using Haupage version 3.3.18038 software and Kappa CF 15/4 MC-S camera (Leica). The MDCK cells were recorded (in CO2 independent media) on the inverted DMIRBE inverted research microscope. The capillary was placed 20 nmover the confluent cells with a constant flow (1300 hPa). The MDCK cells were exposed to peptide and calcein (20 mM) fractions for 60 minutes.

## Results

### HNP 1-3 expression in tissue and serum

**Figure 1:**
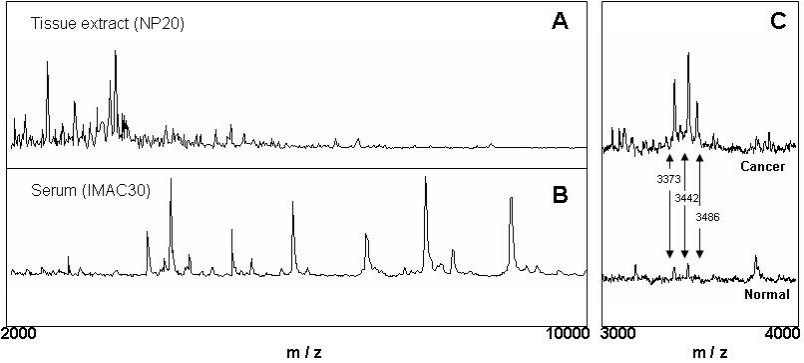
**Protein profiles of serum and tissue. **Representative SELDI-TOF/MS spectra of normal colon tissue (FIG. 1A) on the NP20 chip and normal serum (FIG. 1B) on the IMAC30 (Ni) chip. The two spectra differ significantly and each produce a total of 40 to 60 peaks. The majority of the peaks are present in the specified range from 2 to 10 kDa. FIG. 1C Comparison of a typical colon tumor spectrum (above) and a normal colon spectrum (below) in the range from 3 to 4 kDa. The arrows point to the three differentially expressed peptides, subsequently identified as HNP 1-3. The three peptides are expressed in normal colon tissue and the expression is upregulated in the tumor samples. The three peptides are present in normal serum, but are present in higher concentrations in colon cancer serum. The average peak intensity for the three peptides were significantly lower in serum than in tissue.

**Figure 2:**
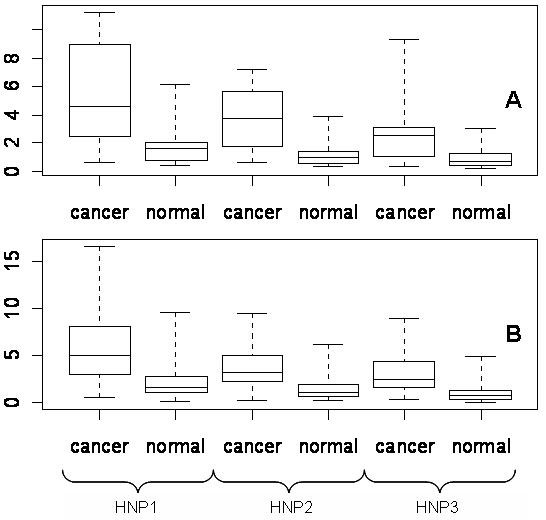
**Expression of HNP 1-3 in tumors and serum. **FIG.2A HNP 1-3 profiles of normal and colon tumor tissue. 40 colon tumor and 40 normal colon tissue samples were analysed on NP20 chips. Differences in mean intensities of HNP 1-3 in normal and colon tumor tissue were statistical significant at 5% level (p < 0.0005). FIG. 2B HNP profiles of normal and colon cancer serum. Serum samples (125 colon cancer and 100 normal) were analyzed on IMAC30 chips. The mean intensities were significantly different at 5% level (p < 2.2e-16). The reproducibility was significant for all three peptides in both tissue and serum. The standard deviation of HNP 1, 2 and 3 was 70%, 136% and 57% in normal tissue and 11%, 15% and 8% in tumor tissue. The standard deviation of HNP 1, 2 and 3 was 96%, 154% and 81% in normal serum and 234%, 365% and 282% in cancer serum. The boxplot show the 25th quantile, median, 75th quantile, and whiskers extent to min. and max. values.

We performed pilot studies of colon tumour and normal colon tissue on a variety of chips with different chemical properties and with different binding and washing conditions. Based on these preliminary studies, we found that the expression of three peptides with mass/charge ratio (m/z) values of 3372, 3443 and 3486 (subsequently identified as HNP 2, 1 and 3, respectively), were upregulated in the tumour samples. The three peptides were visible on different chips and under different binding conditions (data not shown). The strongest signal of HNP 1-3 in tissue extract was obtained on the NP20 (Normal Phase) chip, whereas the strongest signal of HNP 1-3 in serum was observed on the IMAC30 (Ni) (immobilised metal affinity capture chip, activated with nickel), and these conditions were chosen for the actual screenings. We emphasize that in general the protein profiles of serum and tissue were very different when using the same protein chip for serum and tissue extract. However, individual peaks were present on several types of chips, and observed in both serum and in tissue extract, for example the characteristic triplet with m/z 3372, 3443 and 3486. Protein extract from 40 colon tumour and 40 normal colon tissue samples were analysed on NP20 chips and 125 colon cancer serum samples and 100 normal serum samples were analysed on IMAC30 (Ni) chips. All spectres in each screening were pooled and normalised based on overall ion current. Each spectrum produced approximately 40 to 90 protein peaks in the range from 2 to 80 kDa (FIG. 1A–C). Statistical analysis of the intensity values of HNP 1-3 in the tissue screening (FIG. 2A.) showed that HNP 1-3 were significantly upregulated in tumours (p < 0.0005) and statistical analysis of HNP 1-3 expression in the serum screening (FIG. 2B.) showed that HNP 1-3 were significantly upregulated in cancer serum (p < 2.2e^-16^).

Compared to other peptides in the same range, HNP 1-3 showed average signal intensity in most normal colon tissue extract, whereas the HNP 1-3 signal was extremely high in most tumour samples (in some tumour spectres the HNP 1-3 peaks were the strongest of all peaks). In the normal serum samples the HNP 1-3 signals were weak and only slightly stronger in the cancer serum.

### Identification of HNP 1-3

The markers were purified by RP-HPLC, peptide gelfiltration and on-chip purification, after which they were identified by peptide mapping as HNP-2 (3372 Da), HNP-1 (3442 Da) and HNP-3 (3486 Da) (Table 1A). The masses correspond to the peptides in their oxidised states with three disulfide bridges. After reduction with DTT, HNP-1 and HNP-2 increased 6 Daltons in mass, due to reduction of the six cysteines (Table 1B). We were not able to reduce HNP-3.

### Size exclusion chromatography of HNP 1-3

**Figure 3:**
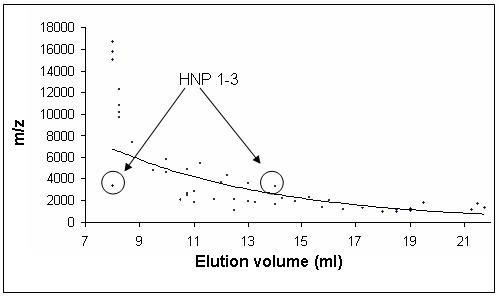
**Size exclusion study of HNP 1-3. **Protein extract from tumor tissue was separated on a peptide gelfiltration column. The elution volumes of forty (unidentified) peptides was plotted against their respective mass values and an approximate elution curve was calculated. The arrows point to HNP 1-3, which were eluted in two fractions: primarily in the void volume (8 ml) together with high mass proteins (above 20 kDa) and after 14 ml together with peptides of similar mass range (2–4 kDa). We interpret this as evidence for binding between HNP 1-3 and high mass proteins.

50 ul colon tumour extract in Lysis buffer was applied to a peptide gelfiltration column. Elution of peptides was followed by absorbance at 280 nm. All fractions were analysed by MALDI-TOF on the PBS II instrument (Ciphergen). Maximum signal intensity of 40 individual peaks was plotted as a function of the elution volume and an approximate elution curve was calculated (FIG. 3). HNP 1-3 peptides were primarily eluted in early fraction together with high mass proteins above 20 kDa and also, but to a lesser degree, in fractions together with other peptides of similar mass interval (2 to 4 kDa) (FIG. 3).

### Cytoxic assay

**Figure 4:**
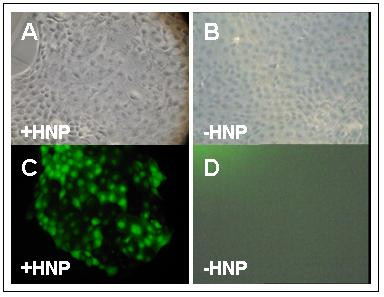
**Functional study of HNP 1-3. **Normal microscopy (A&B) and flourescence microscopy (C&D) of MDCK cells. MDCK cells were exposed to calcein with (A&C) and without HNP 1-3 (B&D). By fluorescence microscopy (C&D) the cells were observed to uptake calcein only when treated with fractions containing HNP 1-3/calcein (C). Fractions containing unidentified peptides purified from colon tumors were used as negative controls together with calcein and did not stimulate the cells to uptake calcein (D). Cell islands treated with HNP 1-3 appeared diffuse and showed enlarged nuclei, indicating apoptosis (A).

The cytotoxicity of HNP 1-3 purified from colon tumours was tested by exposing MDCK cells to different fractions purified from colon tumours. Calcein were added to the fractions and the solutions were left to flow over the cells for one hour. By fluorescence microscopy calcein was observed to accumulate in cells exposed to HNP 1-3/calcein fractions, whereas cells treated with fractions containing other (unidentified) tissue peptides did not uptake calcein (FIG. 4C&D). Further, by microscopy we observed that cells exposed to HNP 1-3 appeared more diffuse and had enlarged nuclei, indicating apoptosis (FIG. 4A&B).

## Discussion

### Elevated concentrations of HNP 1-3 in colon cancer serum

Abnormal concentration of HNP 1-3 in blood has previously been demonstrated in connection with benign conditions. Elevated concentrations of HNP 1-3 following infection (bacterial- / non-bacterial- infection and pulmonary tuberculosis) has been found in plasma, blood and other body fluids [8], and plasma HNP 1-3 concentrations have been shown to be elevated in patients with septicemia or bacterial meningitis [9]. HNP 1-3 have been found in urine from patients with transitional cell carcinoma of the bladder [10] and HNP-1 has been found in salvia of patients with oral carcinomas [11]. Finally, HNP 1-3 are found in excess amounts in tears after ocular surface surgery [12]. Our study is the first that demonstrate elevated concentrations of HNP 1-3 in blood following tumour growth.

### Elevated concentrations of HNP 1-3 in colon tumours

HNP expression has previously been linked to different types of tumours and cell lines. HNP-1 has been detected in lung tumours [13] and in the submandibular glands of patients with oral carcinomas [14]. By RT-PCR, mass spectrometry and flow cytometric analysis, HNP 1-3 have been shown to be expressed by cell lines deriving from renal cell carcinomas [15] and the expression of a specific HNP precursor peptide has been shown to be upregulated in human leukemic cells [16]. Our results suggest that HNP 1-3 are extremely abundant in colon tumours. This is in agreement with a study of HNP-1 in lung tumours, where the maximum observed level was 26 nanomoles per gram wet tissue [13]. In order for these excessive amounts of peptide to be detectable in serum, the peptides must be present extracellular in the tumour environment, such as observed in studies of HNP 1-3 expression in kidney [14] and brain [17]. Usually tumour expressed peptides are not easily detected in serum or plasma by SELDI-TOF mass spectrometry techniques; the highly concentrated plasma proteins out compete the signal of low abundance peptides. We suggest that HNP 1-3 are detectable in serum only because they are expressed in exceptionally high amounts in the tumour microenvironment.

Previous studies indicate that HNP 1-3 expression in tumours primarily originate from tumour invading eosinophils [13] and neutrophils [14,18]. However, it has been shown that the excess amounts of HNP 1-3 observed in urine from bladder cancer patients were often produced by the actual bladder cancer cells [10], and that highly invasive bladder cancer cells produced more HNP 1-3 than less invasive ones (Holterman DA, in press, personal communication). Since our tissue screening is based on comparison of tissue samples, we cannot say whether the peptides are produced by the colon cancer cells or by tumour infiltrating neutrophils. In the case of active inflammatory bowel disease, it is not clear whether epithelial expression of HNP1-3 is induced by the inflammatory state or the whether the peptides are released by adjacent neutrophils and taken up by the epithelial cells [6]. Here it is believed that the peptides provide protection against microbial invasion when the mucosal barrier is damaged (such as is the case in inflammatory bowel diseases). HNP 1-3 are known to stimulate bronchial epithelial cells to upregulate interleukin-8 production [19], a potent neutrophil chemotactic factor. Thus, the upregulated expression of HNP 1-3 in tumours may primarily originate from invading immune cells, but could be initiated by HNP 1-3 producing cancer cells.

### Size exclusion chromatography of HNP 1-3

We explain the elevated concentrations of HNP 1-3 in colon cancer serum by unspecific binding between HNP 1-3 and high mass plasma proteins. We suggest that the peptides attach to plasma proteins in the tumour area and are carried into the bloodstream. The HNP 1-3 we observe in high mass fractions from size exclusion, could also be explained by multimerisation: In one study it was demonstrated that defensins form voltage dependent channels in lipid bilayer membranes and further conductance investigations suggested that the channels were formed by multimers containing 2–4 molecules [20] and a crystal structure study [21] of HNP-3 revealed an amphiphilic dimmer. We interpret the size exclusion results as evidence for interaction between HNP 1-3 and unidentified high mass proteins through unspecific interactions; the peptides are eluted in very early fractions which would probably require the presence of multimers of more than five peptides and our study does not reveal the presence of multimers containing four, three or two peptides. Further, our interpretation is in agreement with a number of previous studies that show that HNPs are bound to plasma protein *in vitro *and that high concentrations of HNPs causes precipitation of plasma proteins; specifically 2-macroglubulin and C1 complement [22,23] has been shown to bind defensin. Another study [24] showed that HNP-1 bind to various plasma proteins, notably serum albumin, and it was found that serum, or serum albumin, was able to inhibit the anti-viral activity of HNP-1. This ability to bind to plasma proteins could also explain why HNP 1-3 lysis of mammalian cells is hindered in the presence of serum [25]. Together these observations add to the growing realisation that common plasma proteins may carry disease specific peptides and therefore should not be ignored in biomarker research.

Common to beta-defensin 2 (another member of the defensin family) and HNP 1-3 is an uneven distribution of surface charges. Beta-defensin 2 has been shown to bind to a chemokine receptor [26]. It has been suggested that the positively charged cluster found in defensin peptides and chemokines, may play a common role in binding to receptors, but is not important for determining receptor specificity [27]. This surface charge may also explain the binding of HNP 1-3 to plasma proteins. The observation that defensins are localised to lymphocyte nuclei [28], could similarly be explained by unspecific binding to shuttle proteins.

### The function of HNP 1-3

The exact concentration of HNPs in the tumour microenvironment may decide the *in vivo *function of HNP 1-3. One study showed that HNP 1-3 mediates lysis of tumours in a concentration dependent manner [25]. This is in agreement with another study that show that only relatively high concentrations of HNP-1 (10^-4 ^M) are cytotoxic for human monocytes, whereas lower concentration of HNP-1 (10^-8 ^to 10^-9 ^M) increases TNF-alpha production by monocytes [29]. In a study of renal cell carcinoma lines [14] it was shown that HNP 1-3 were cytotoxic to all tested cell lines when present in high concentrations (above 25 ug/ml), but at lower concentration HNP 1-3 stimulated growth of a subset of tumour cell lines. We add to these results by demonstrating that *in vivo *HNP 1-3 purified from colon tumours are capable of lysing MDCK cells. Our study was based on a 60 minutes microflow study. This screening set-up did not allow us to investigate the minimum concentration of HNP 1-3 necessary for lysis.

## Conclusions

The high concentration of HNP 1-3 observed in tumours and the observation that HNP 1-3 are capable of lysing mammalian cells may lead to the conclusion that the peptides serve to the benefit of the host by killing tumour cells. However, in one study HNP 1-3 were found to bind to HLA-Class II molecules and were capable of reducing the proliferation of a HLA-DR-restricted T-cell line after stimulation [30] and could in this way help the cancer cells avoid local immune recognition. Defensins also regulate the systemic immune response. Through interaction with the chemokine receptor CCR6, beta-defensins recruits dendritic and T cells[26] (discussed in [31]) and HNP 1-3 are capable of recruiting leukocytes to sites of infection in mice [32]. Upregulated immune responses are known to stimulate tumour proliferation; immune cells are actively recruited by tumours to exploit their pro-angiogenic and pro-metastatic effects (reviewed in [33,34]). Whether the high concentrations of HNP 1-3 in the tumour limits the tumour growth or on the contrary stimulate tumour proliferation is not clarified; we emphasise that HNP 1-3 are expressed in inflammatory bowel disease and could be involved in the early stages of carcinogenesis. We suggest that the prominent surface charge on defensins, their unspecific binding to other proteins (such as high mass plasma proteins) and the observed excess amounts of peptides found in tumours, could provide the peptides with broad antagonising effects on numerous receptors in the tumour microenvironment. In this way HNP 1-3 peptides may serve to the benefit of the tumour.

Our results add to the evidence that HNP 1-3 bind to high mass plasma proteins. We suggest that the peptides are released in the inflammatory site and passively diffuse into the blood stream. HNP 1-3 have been observed in primary tumours of different tissues and the peptides are known to play a fundamental role in the innate immune system. We suggest that the peptides may serve as markers for colon cancer in combination with established diagnostic tools and as prognostic markers following therapy.

## Competing interests

This study was partly financed by Colotech ltd. and partly by Glostrup Hospital. Jakob Albrethsen, Rikke Bøgebo and Hans Raskov receive salary from Colotech A/S.

Steen Gammeltoft, Jesper Olsen and Benny Winther receive salary from Glostrup Hospital.

## Authors' contributions

JA performed the SELDI-TOF/MS screenings, the protein identification experiments and the size exclusion study. RB did the statistical analysis, BW did the microflow study, JO obtained the biological samples. HR and SG planned the project.

## Pre-publication history

The pre-publication history for this paper can be accessed here:



## Figures and Tables

**Table 1:** Identification of HNP 1-3. Table 1A HNP 1-3 peptides were identified by peptide mapping (on-chip trypsin digest). Three sequence specific fragments were produced (615.8, 930.6, 1061.4 Da.). The peptides were identified as HNP 1-3 using the PepIdent server on the ExPASy homepage. Table 1B The measured masses of the HNP 1-3 peptides were approximately 6 Daltons lower than the expected theoretical masses. This deviation was caused by the *in vivo *formation of three disulfide bridges. By heat denaturation and treatment with DTT we were able to protonate the six cysteines in HNP 1 and 2. (HNP-3 was consistently degraded during the reduction procedure).

**A**			
**Measured masses from peptide mapping (Dalton)**	**Matching theoretical masses (Dalton)**	**Deviation (Dalton)**	**Peptide sequence**
615.8	615.7	+0.1	ACYCR
930.6	930.1	+0.5	IPACIAGE
1061.4	1061.2	+0.2	YGTCIYQ

**B**			
**Measured masses of HNP 1-3 peptides (Dalton)**	**Theoretical masses of HNP 1-3 peptides (Dalton)**		**Measured masses of reduced HNP 1-3 peptides (Dalton)**

3371.2	3377.0 (HNP-2)		3376.2
3442.3	3448.1 (HNP-1)		3448.6
3486.3	3292.1 (HNP-3)		-
